# Acute Stress-Induced Blood Lipid Reactivity in Hypertensive and Normotensive Men and Prospective Associations with Future Cardiovascular Risk

**DOI:** 10.3390/jcm10153400

**Published:** 2021-07-30

**Authors:** Cathy Degroote, Roland von Känel, Livia Thomas, Claudia Zuccarella-Hackl, Jens C. Pruessner, Roland Wiest, Petra H. Wirtz

**Affiliations:** 1 Biological Work and Health Psychology, University of Konstanz, 78457 Konstanz, Germany; cathy.degroote@uni-konstanz.de (C.D.); livia.thomas@ibp-institut.ch (L.T.); 2 Department of Consultation-Liaison Psychiatry and Psychosomatic Medicine, University Hospital Zurich, University of Zurich, 8091 Zurich, Switzerland; Roland.VonKaenel@usz.ch (R.v.K.); Claudia.Hackl-Zuccarella@usz.ch (C.Z.-H.); 3 Clinical Neuropsychology, University of Konstanz, 78457 Konstanz, Germany; jens.pruessner@uni-konstanz.de; 4 Centre for the Advanced Study of Collective Behaviour, University of Konstanz, 78457 Konstanz, Germany; 5 Support Center of Advanced Neuroimaging, Institute of Diagnostic and Interventional Neuroradiology, University Hospital Bern, University of Bern, 3010 Bern, Switzerland; Roland.Wiest@insel.ch

**Keywords:** blood lipid stress reactivity, essential hypertension, Montreal Imaging Stress Task, cardiovascular risk, TC/HDL-C ratio, interleukin-6, D-dimer, hemoglobin A1c

## Abstract

Hyperreactivity to stress may be one explanation for the increased risk of cardiovascular disease (CVD) in individuals with essential hypertension. We investigated blood lipid reactivity to the Montreal Imaging Stress Task (MIST), a psychosocial stressor, in hypertensive and normotensive men and tested for prospective associations with biological risk factors. Fifty-six otherwise healthy and medication-free hypertensive and normotensive men underwent the MIST. We repeatedly measured cortisol and blood lipid profiles (total cholesterol (TC), low-density lipoprotein cholesterol (LDL-C), high-density lipoprotein cholesterol (HDL-C), and triglycerides (TG)) immediately before and up to 1 h after stress. Lipid levels were corrected for stress hemoconcentration. Thirty-five participants completed follow-up assessment 2.9 ± 0.12 (*SEM*) years later. CVD risk was assessed by prospective changes in TC/HDL-C ratio, IL-6, D-dimer, and HbA1c from baseline to follow-up. The MIST induced significant changes in all parameters except TC (*p*-values ≤ 0.043). Compared with normotensives, hypertensives had higher TC/HDL-C-ratio and TG (*p*-values ≤ 0.049) stress responses. Blood lipid stress reactivity predicted future cardiovascular risk (*p* = 0.036) with increases in HbA1c (ß = 0.34, *p* = 0.046), IL-6 (ß = 0.31, *p* = 0.075), and D-dimer (ß = 0.33, *p* = 0.050). Our results suggest that the greater blood lipid reactivity to psychosocial stress in hypertensives, the greater their future biological CVD risk. This points to lipid stress reactivity as a potential mechanism through which stress might increase CVD risk in essential hypertension.

## 1. Introduction

Cardiovascular diseases (CVD) rank among the leading causes of death in adulthood [1], with hypertension being one major risk factor [2]. Most hypertensive patients are diagnosed with essential hypertension as the medical cause for their chronically elevated blood pressure (BP) is unclear [3,4]. Hypertension often occurs in conjunction with other cardiovascular risk factors such as older age, obesity, insulin resistance, diabetes, and dyslipidemia [2,4]. Especially dyslipidemia has frequently been observed in hypertensive individuals compared to normotensive controls, including increased total cholesterol (TC), triglycerides (TG), and low-density lipoprotein cholesterol (LDL-C) on the one hand, and decreased high-density lipoprotein cholesterol (HDL-C) on the other [5,6,7]. This adverse lipid profile is reflected by an excess proportion of atherogenic lipoproteins over HDL-C in hypertensives [8,9]. In particular, the TC/HDL-C ratio has been described as a suitable index reflecting the magnitude of dyslipidemia [10] with a strong predictive value for the incidence of both ischemic heart disease and cardiovascular risk [10,11].

Mental stress is an independent psychological risk factor for CVD [12]. According to the stress reactivity hypothesis, the study of physiological responses to controlled short-term challenges yields important insights into the complex psychobiological processes involved in the development of CVD [13]. Different acute mental stressors have been shown to elicit transient elevations in atherogenic lipids and the TC/HDL-C ratio [14,15,16,17,18,19], although only few studies found stress-induced lipid increases independent of stress hemoconcentration [15,16,17]. This is important because hemoconcentration confounds measurement of stress-induced changes in blood lipids [20]. More precisely, stress can induce transient acute loss of plasma volume into the extravascular space, which results in concentration and passive increase of nondiffusible blood constituents such as lipids [18]. While studies using mild stressors did not show blood lipid increases independent of hemoconcentration [18,19], studies with more potent stressors including psychosocial threat elements did so [15,16,17]. For instance, applying the Trier Social Stress Test (TSST) [21], which combines public speaking and mental arithmetic to be delivered in front of an audience, we found increased TC and LDL-C responses in hypertensives compared to normotensives [17]. These results suggest hyperreactivity of atherogenic lipids to stress in hypertensives. Similar to the TSST, the Montreal Imaging Stress Task (MIST) is a psychosocial stressor that comprises motivated performance with social evaluative threat and uncontrollability [22]. So far, the MIST has been shown to reliably induce substantial increases in salivary cortisol indicative of hypothalamic–pituitary–adrenal axis (HPA) activation as well as increases in blood pressure and heart rate indicating sympathoadrenal medullary (SAM) arousal [22,23]. Thus far, studies on blood lipid reactivity using the MIST have not been performed.

The responsivity of blood lipids to an acute standardized stressor might contribute to pathophysiological processes involved in the development of CVD. For instance, greater lipid responses to acute mild stress predicted higher TC/HDL-C ratios several years later in London-based civil servants, although stress hemoconcentration effects were not accounted for [24]. To our best knowledge, there are no studies that have investigated prospectively the effects of blood lipid stress reactivity on intermediate biomarkers of an increased CVD risk, reflecting low-grade systemic chronic inflammation (interleukin (IL)-6 [25]), a prothrombotic state (fibrin D-dimer [26]), or a diabetic condition (glycosylated hemoglobin A1c, HbA1c [27]).

We aimed to investigate (1) whether acute psychosocial stress induced by the MIST would induce changes in blood lipid levels independently of hemoconcentration in hypertensive and normotensive participants. We hypothesized that hypertensives would show greater stress-induced increases in TC, LDL-C, TG, and the TC/HDL-C ratio, but reduced HDL-C compared with normotensives. Moreover, (2) to shed light on the potential clinical relevance of higher blood lipid stress reactivity, we investigated whether reactivity of the TC/HDL-C ratio to the MIST would predict IL-6, D-dimer, and HbA1c levels after a follow-up of 2 to 5 years. We hypothesized higher blood lipid stress reactivity to predict future increases in these biomarkers of atherothrombotic CVD risk.

## 2. Materials and Methods

### 2.1. Study Participants

The current investigation is part of a series of studies assessing psychoneurobiological mechanisms in essential hypertension [28,29] approved by the ethics committee of the State of Bern, Switzerland. All participants provided written informed consent and were financially compensated with 140 CHF.

With the aid of the Swiss Red Cross of the State of Bern, we recruited apparently healthy, nonsmoking hypertensive and normotensive men of comparable age. In detail, members of our study team accompanied the mobile blood-donation unit of the Swiss Red Cross that routinely records BP before blood donation. Blood donors interested in the study participation were given written information asking for the following inclusion criteria: age between 18 and 80 years; BP either in the hypertensive or in the normotensive range (see below); nonsmoker (<5 cigarettes per day), and no alcohol or illicit drug abuse. Specific exclusion criteria were verified by telephone interview using an extensive health questionnaire [28,29]. Participants were not eligible to participate when they reported any current infectious disease, current use of medication, a diagnosis of heart disease, elevated cholesterol, elevated blood sugar and diabetes, liver and renal diseases, chronic obstructive pulmonary disease, rheumatic diseases, HIV, cancer, chronic or acute clinical psychiatric disorders, as well as regular strenuous physical exercise, tobacco consumption, excessive alcohol, and illicit drug abuse. Four eligible participants (one normotensive and three hypertensives) who reported regular medication intake stopped taking their medication 1 week prior to the study. Eligible hypertensive participants provided blood samples for the routine assessment of serum creatinine, calcium, sodium, and potassium to exclude potential cases with secondary hypertension. Due to technical problems, sodium, potassium, and calcium could not be analyzed in five hypertensive participants. In one of these participants, creatinine could not be measured either. No eligible hypertensive participant was diagnosed with secondary hypertension. We measured HbA1c and blood lipids (see below) in all participants.

### 2.2. Classification of Essential Hypertension and Normotension

Classification of essential hypertension and normotension was based on a two-step assessment procedure. In step 1, following written instructions, interested blood donors were asked to measure their BP on three days at home using sphygmomanometry (Omron M6; Omron Healthcare Europe B.V., Hoofdorp, Netherlands). Home BP was to be self-measured once in the morning and once in the evening in a seated position, each after a 15-min rest. Participants were recruited as hypertensive if the average home systolic blood pressure (SBP) was ≥135 mmHg and/or the average home diastolic blood pressure (DBP) was ≥85 mmHg according to recommendations for home BP measurements [30]. Correspondingly, participants were recruited as normotensives if the average home SBP was <135 mmHg and the average home DBP was <85 mmHg. We computed the average home BP of the six measurements obtained by each participant. Home BP measurement was provided by a total of 63 potential participants. Two participants were diagnosed with essential hypertension prior to their study participation and did not perform home measurements.

In step 2, we verified the home-measurement-based preliminary classification of each of these participants as hypertensive or normotensive. Trained personnel performed three additional BP measurements during the first study session with the participant in a seated position after a 15-min rest. We applied the regular World Health Organization (WHO)/International Society of Hypertension (ISH) definition of hypertension and classified medication-free participants as hypertensive if their average study SBP was ≥140 mmHg and/or their average study DBP was ≥90 mmHg [31]. Participants were classified as normotensive if their average study SBP was <140 mmHg and their average study DBP was <90 mmHg.

The final group assignment of medication-free participants was based on congruent home and study BP classification and comprised 28 normotensives and 28 essential hypertensives. Six participants were excluded due to normotensive home, but hypertensive study BP (white coat hypertension), and three participants were excluded due to hypertensive home and normotensive study BP (masked hypertension).

### 2.3. Design and Procedure

Cross-sectional data assessment comprised 2 days. On the 1st study day, we performed study BP classification to verify the home-measurement-based preliminary classification of hypertension and normotension and to assess medical information. Hypertensive and normotensive participants with congruent home and study BP classification were then invited to a 2nd study day to undergo a standardized stress test.

#### 2.3.1. First Study Day and Baseline CVD Risk Assessment

All participants consumed a semistandardized breakfast following written instructions and abstained from caffeine and alcohol consumption for 24 h prior to their arrival at the lab at 8:00. After providing written informed consent, participants´ body measurements (height and weight) were assessed. Resting study BP was assessed three times by means of sphygmomanometry (Omron M6; Omron Healthcare Europe B.V., Hoofdorp, Netherlands), each after a 15-min rest period. Blood samples were collected at 11:30, i.e., after a fasting for 3.5 h since arrival. Due to technical problems, baseline blood sampling was missing in one hypertensive participant and incomplete in one normotensive participant.

#### 2.3.2. Second Study Day and Stress Reactivity Assessment

All participants were instructed to abstain from food for at least 1 h, and from alcoholic and caffeinated beverages and strenuous exercise for at least 24 h before the second study appointment. They were further required to be well rested and to have maintained a regular sleep–wake rhythm for three nights before the reactivity testing. Participants arrived between 12:00 and 13:00 at the lab, where they received detailed explanations of the testing procedure and provided informed consent for the 2nd study day. Then, trained study nurses inserted an intravenous catheter into the participants’ nondominant forearm for blood sampling. During the following 45-min acclimatization period, participants completed psychosocial questionnaires ahead of the collection of baseline blood samples. Afterwards, participants underwent a standardized psychosocial laboratory stressor for imaging studies (i.e., the MIST) as explained below. The recovery period started after cessation of the stressor and lasted for 1 h. Blood and saliva samples were collected immediately before stress induction by the MIST (−1 min) and repeatedly after MIST cessation (+1 min, +10 min, +20 min, and +60 min). Additional saliva samples were assessed 30 and 45 min after stress. Participants were in supine position during the experimental procedure, and all blood samples were drawn in supine position. Participants only raised to move from the test room to the nearby scanner room (after MIST baseline blood sample) and back again (after blood sample +1 min). They were also unrestricted to visit the restrooms at any time.

#### 2.3.3. Assessment of CVD Risk at Follow-Up

To assess longitudinal changes in CVD risk factors, we invited our study participants to a follow-up assessment at least 2 years later for blood sampling procedures identical to the 1st study day. All 56 participants of the baseline assessment were invited for the follow-up assessment scheduled between 2 and 5 (*M* = 2.86 ± 0.12) years later, with a final sample of 35 participants completing both assessments. Reasons for drop-out comprised severe illness (*n* = 4), no interest (*n* = 2), not reachable by phone (*n* = 5), lack of time (*n* = 5), excessive demand (*n* = 1), or no reason given (*n* = 4). Notably, we initially intended to schedule for a 2-year follow-up period. However, due to the relocation of the working group from Bern (Switzerland) to Konstanz (Germany), the follow-up assessments in Bern were hampered by limited personnel resources and logistic challenges. This resulted in substantially longer follow-up intervals with potential effects on drop-out rates.

### 2.4. Montreal Imaging Stress Task (MIST)

Psychosocial stress was induced following the standard protocol of the MIST. This standardized stress paradigm was developed for functional imaging studies and reliably induces psychophysiological stress responses [22]. The MIST is based on the Trier Mental Challenge Task, and combines a series of computerized mental arithmetic challenges with social evaluative threat components [32]. The MIST was carried out with (stress condition) or without (control condition) time pressure and social evaluation.

In detail, starting with a training session, participants’ ability to perform mental arithmetic was assessed outside the scanner, and the average time needed to solve problems was used to set a default time limit for the experimental condition. In both conditions (stress and control), participants had to solve mental arithmetic tasks presented on a projection screen, which additionally displayed performance feedback (i.e., correct, incorrect, or time out). Stress was induced by consistently adapting the difficulty and time provided to solve the mental arithmetic to ensure a 50% to 60% failure rate. Simultaneously, a continuous performance progress bar on the screen indicated to the participants that their performance was weaker compared to that of all other participants of similar age, educational level, and professional position. Between runs, two confederates, introduced as study investigator and as study leader, enforced social evaluative threat by giving standardized fake feedback [22].

As first feedback, the study investigator reminded the participant that there is a required minimum performance for the participant to be used in the analysis of the functional scan. The investigator informed the participant that the scanning session had to be stopped because of his poor performance. The investigator also asked about potential reasons for this poor performance (e.g., problems with understanding the task, problems with the response box, or visual problems with the screen). Finally, the participant was told that the study investigator, the study leader, and the MR technicians were following his performance on a second monitor in the control room of the scanning environment and asked him to give his best to provide useful data. The investigator then left the scanner room, and the next run of mental arithmetic was initiated.

The second feedback was given by the supposed leader of the study. The latter informed the participant that his performance had been monitored and that the scanning session had to be stopped again because of his poor performance. The study leader asked the participant if he was aware of his below-average performance. He reminded the participant that his performance should at least approach the average user level if his data were to be used in the study. Since he had often exceeded the time provided to solve the mental arithmetic (as indicated by “timeout”), the participant was also asked about potential problems with concentration in the last days or any alcohol intake during the last 24 h. Finally, the participant was asked to try his best to provide useful data; the investigator further explained that useful data were naturally the primary goal of the whole study team since a lot of energy and money had already been spent on the participants’ fMRI data acquisition. Then, the third run of mental arithmetic was initiated.

The control condition consisted of mental arithmetic tasks with comparable difficulty level but presented without any time restriction and/or negative feedback. Individual and average users’ performance were not displayed.

In total, the participants underwent three runs. In each run, the stress (105 s) and the non-stress (45 s) conditions were presented in a blocked design with three repetitions in a counterbalanced order. The two feedbacks were given between runs 1 and 2 (feedback 1), and between runs 2 and 3 (feedback 2). The total duration of the MIST was about 30 min.

### 2.5. Biochemical Analyses

#### 2.5.1. Blood Lipids

Blood lipid profiles were assessed on all three study days. On study day 2, measurements were immediately before and after stress, as well as 10, 20, and 60 min after stress cessation (−1 min, +1 min, +10 min, +20 min, and +60 min). We repeatedly measured total TC and HDL-C to allow computation of TC/HDL-C ratio [10], as well as TG from heparine-coated monovettes (Sarstedt monovette orange). Analyses were performed at the Center for Laboratory Medicine of the Bern University Hospital (Inselgruppe AG, Bern, Switzerland) using in vitro assays (enzymatic colorimetric) for the quantitative determination of blood lipids in human plasma (Roche, Mannheim, Germany) on a Roche/Hitachi Cobas C Analyzer (Roche, Mannheim, Germany). LDL-C was calculated using the Friedewald formula: LDL-C = TC − HDL-C − (TG/2.19). Mean inter- and intra-assay CVs were ≤1.2% and ≤2.5%, respectively. In order to correct plasma lipid levels for stress hemoconcentration, we additionally assessed hemoglobin (grams per deciliter) and hematocrit (percentage) by processing whole blood collected in 2.7 mL EDTA-coated tubes (Sarstedt, Nümbrecht, Germany) on an automated hematology system (Advia 120, Siemens Diagnostics, Erlangen, Germany) at the Center for Laboratory Medicine. Due to technical problems with blood sampling, baseline (i.e., day 1) blood lipids were missing in one hypertensive and one normotensive participant.

#### 2.5.2. Cortisol

We measured cortisol as a manipulation check to test whether the MIST successfully induced cortisol stress reactivity as observed in previous research [22]. For assessment of cortisol, saliva samples were collected at 7 time points (−1 min, +1 min, +10 min, +20 min, +30 min, +45 min, and +60 min) using Salivette devices (Sarstedt, Rommelsdorf, Germany), which were stored at −20 °C until biochemical analysis. Prior to analyses, saliva samples were thawed and spun at 3000 rpm for 10 min, yielding low-viscosity saliva. Cortisol concentrations were measured using a commercially available competitive chemiluminescence immune assay with high sensitivity of 0.16 ng/mL (LIA, IBL Hamburg, Germany). Intra- and inter-assay CVs were <7.7% and 11.5%, respectively. Cortisol assessment was missing in one hypertensive participant, and cortisol data of another normotensive participant were not considered due to an outlier value (>70 nmol/L) at baseline.

#### 2.5.3. CVD Risk Assessment

CVD risk was assessed in all participants at baseline and prospectively at follow-up. We assessed CVD risk by measurement of the following risk factors: TC/HDL-C ratio, the hypercoagulability marker D-dimer, HbA1c, as well as the pro-inflammatory cytokine IL-6. Blood lipids, D-dimer, and HbA1c were analyzed at the Center for Laboratory Medicine of the Bern University Hospital (Inselgruppe AG, Bern, Switzerland), while IL-6 analyses were performed in the biochemical laboratory of the Biological Work and Health Psychology group at the University of Konstanz.

For assessment of D-dimer, venous blood was drawn into polypropylene tubes containing 3.8% sodium citrate (Sarstedt, Nümbrecht, Germany). Citrate tubes were immediately centrifuged for 20 min at 4 °C at 2000× *g*, and plasma was pipetted into aliquots. D-dimer was analyzed using a particle-enhanced immunoturbidimetric assay for the quantitative determination of D-dimer in human plasma (INNOVANCE^®^ D-Dimer, Siemens Healthcare GmbH, Erlangen, Germany) on a Sysmex CS-5100 (Sysmex Europe, Norderstedt, Germany). The intra- and inter-assay coefficients of variation were ≤7.9%.

To measure HbA1c, venous blood was drawn into EDTA-coated monovettes, and analyses were performed with in vitro assays for the quantitative determination of HbA1c IFCC (mmol/mol) in whole blood (Tina-quant^®^, Roche, Mannheim, Germany) using Roche/Hitachi Cobas C Systems (Roche, Mannheim, Germany). Mean inter- and intra-assay CVs were ≤1.6% and ≤2.0%, respectively.

For the determination of IL-6, venous blood was drawn in EDTA-coated monovettes (Sarstedt, Nümbrecht, Germany) and immediately centrifuged for 10 min at 2000× *g* and 4 °C. Obtained plasma was stored at −80 °C until analysis. IL-6 levels were determined with a high-sensitivity sandwich immunoassay (Meso Scale Discovery (MSD), Rockville, MD, USA). Mean inter- and intra-assay CVs were ≤7.3% and ≤4.5%, respectively, and the detection limit was 0.06 pg/mL. Baseline measurements were incomplete in one hypertensive and one normotensive participant (see Table 1 for details).

### 2.6. Statistical Analyses

Statistical analyses were performed using SPSS (Version 26.0) statistical software packages for MacIntosh (IBM SPSS Statistics, Chicago, IL, USA). All tests were two-tailed with level of significance at *p* < 0.05 and *p*-values < 0.10 interpreted as borderline significant.

G*Power (Version 3.1.9.6; Heinrich Heine University Düsseldorf, Germany) analysis suggests that a total sample size of *n* = 54 is needed to detect group differences in plasma lipid stress reactivity (5 repetitions) with a small-to-medium effect size of *f* = 0.15 in general models with repeated measures with a power of 0.85, α = 0.05, given the previously observed minimum intercorrelation among repeated measures of 0.63 and ε = 0.79 [33].

We corrected all plasma lipid levels for stress hemoconcentration following previous methods by computing stress-induced changes in plasma volume (i.e., stress hemoconcentration) from hemoglobin and hematocrit measures according to the formula by Dill and Costill [34,35]. Body mass index (BMI) was calculated by the formula BMI = kg/m^2^.

Univariate analyses of variance (ANOVAs) were used to compute group differences in participant characteristics. To test for MIST-induced increases in the studied measures, we calculated ANOVAs with repeated measures for cortisol (baseline, 1 min, 10 min, 20 min, 30 min, 45, min, and 60 min post-stress) and blood lipids (baseline, 1 min, 10 min, 20 min, and 60 min post-stress) over all participants. Significant changes of each measurement from baseline were identified post hoc.

As main analyses, we first investigated whether hypertensives exhibited higher blood lipid increases to acute stress compared with normotensives. We calculated repeated-measures ANCOVAs with repeated assessment of hemoconcentration-corrected blood lipids (baseline, 1 min, 10 min, 20 min, and 60 min post-stress) as dependent variables and with group as the independent variable. Repeated measures ANCOVAs were also calculated to examine possible group differences in cortisol (baseline, 1 min, 10 min, 20 min, 30 min, 45, min, and 60 min post-stress). We controlled for age and BMI as covariates in the repeated-measures ANCOVAs. Post hoc testing of significant stress effects in cortisol and blood lipid measures comprised univariate ANOVAs with group as the independent variable and the respective single measurements as dependent variables.

We performed prospective analyses to shed light on the potential clinical relevance of blood lipid stress reactivity and analyzed whether blood lipid stress responses would predict future changes in CVD risk factors. We calculated multiple analyses of variance (MAN(C)OVA) with aggregated blood lipid stress reactivity calculated as area under the curve with respect to ground (AUC_G_) [36] as independent variable. Dependent variables were prospective changes in TC/HDL-C ratio, D-dimer, HbA1c, and IL-6 from baseline to follow-up assessment (follow-up measurement of the respective parameter minus its baseline measurement) to investigate CVD risk as dependent variable. Baseline age, MAP, and time between baseline and follow-up assessments were controlled as a set of covariates in complementary analyses, in addition to BMI, prospective changes in BMI and MAP, or medication intake at follow-up assessment. Post hoc testing comprised linear regression analyses with the aggregated blood lipid stress reactivity (AUC_G_) as independent variable and changes in CVD risk factors separately (i.e., changes in TC/HDL-C ratio, D-dimer, HbA1c, and IL-6) as dependent variables, while controlling for the set of confounders, i.e., covariates.

All data were tested for normal distribution and homogeneity of variance using Kolmogorov–Smirnov and Levene’s tests prior to statistical analyses. All measures showing a skewed distribution (home MAP and SBP, stress reactivity of cortisol and most TG levels, HbA1c, IL-6, and D-dimer at baseline as well as prospective changes in TC/HDL-C ratios, HbA1c, IL-6, BMI, and MAP) were log-transformed. While log-transformed data were used for modeling and testing, we depict untransformed data in all Tables and Figures for reasons of clarity. Blood lipids stress reactivity was depicted as (percentage) changes from baseline to post-stress.

We applied Huynh–Feldt correction for repeated measures where appropriate. We determined *f* from partial η^2^ values using G*Power. Effect size parameters *f* (effect size conventions *f*: 0.10 = small; 0.25 = medium; 0.40 = large) and *R*^2^ changes (effect size conventions Δ*R*^2^: 0.02 = small; 0.13 = medium; 0.26 = large) are reported where appropriate [37].

## 3. Results

### 3.1. Group Characteristics

Table 1 shows the demographic, medical, and psychological characteristics of the 28 hypertensive and 28 normotensive participants. As expected, hypertensives showed higher systolic and diastolic BP as well as MAP, higher BMI, IMT, IL-6, and fasting TG, as well as higher LDL-C/HDL-C and TC/HDL-C ratio at baseline (i.e., on the first study day) compared with normotensives (*p*-values ≤ 0.010).

Hypertensives had also a higher MIST baseline TC/HDL-C-ratio than normotensives (*p* = 0.023) did, but there were no other differences in MIST baseline blood lipid levels (*p*-values ≥ 0.11; see Table 2). At follow-up, eight of the initially medication-free participants were under medication: five hypertensives and one normotensive took BP-lowering medication, one hypertensive took cholesterol-lowering medication, and another hypertensive participant took uric acid-lowering medication.

Participants who dropped out (*n* = 21) did not significantly differ in any group characteristic (*p*-values ≥ 0.15; see Appendix A, Table A1) from participants completing both baseline and follow-up assessment (*n* = 35).

### 3.2. Cortisol and Blood Lipid Reactivity to the MIST in All Participants

#### 3.2.1. Cortisol

**Figure 1:**
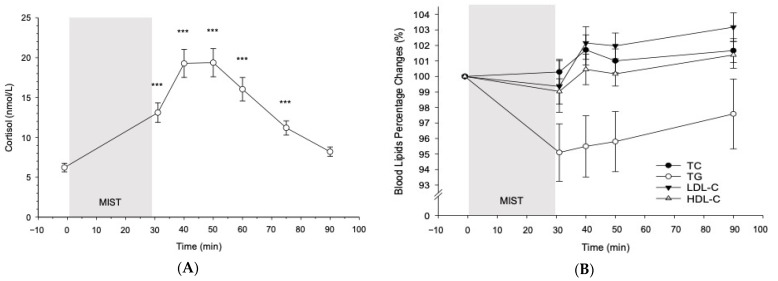

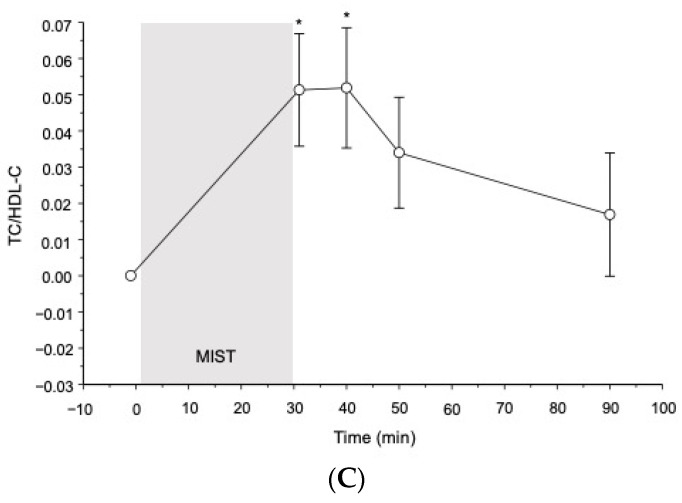
(**A**–**C**). Cortisol and blood lipid changes in response to the MIST. Values are means ± *SEM*. (**A**) Cortisol. (**B**) Percentage changes from baseline to post-stress in TC, TG, LDL-C, and HDL-C. (**C**) TC/HDL-C ratio changes from baseline in response to MIST. Asterisks indicate significant differences between baseline and the respective post-stress levels * *p* < 0.05; *** *p* < 0.001.

As a manipulation check, we tested whether the MIST successfully induced cortisol stress reactivity as observed in previous research [22] (see Figure 1A). Across all participants, the MIST induced significant increases in cortisol (main effect of stress: *F*(2.88, 152.86) = 70.39, *p* < 0.001, partial η^2^ = 0.57, *f* = 1.15), with highest levels observed 10 min after MIST cessation. Post hoc testing revealed significant increases from MIST baseline to 1 min (*p* < 0.001), 10 min (*p* < 0.001), 20 min (*p* < 0.001), 30 min (*p* < 0.001), and 45 min (*p* < 0.001) after MIST cessation.

#### 3.2.2. Blood Lipids

As shown in Figure 1B, the MIST induced in all participants significant increases in HDL-C (main effect of stress: *F*(3.50, 192.70) = 2.62, *p* = 0.043, partial η^2^ = 0.05, *f* = 0.22) and LDL-C levels (main effect of stress: *F*(2.95, 161.96) = 3.53, *p* = 0.017, partial η^2^ = 0.06, *f* = 0.25), while increases in TC levels did not reach statistical significance (*p* = 0.13). In contrast, TG levels decreased in all participants (main effect of stress: *F*(2.0, 109.70) = 5.01, *p* = 0.008, partial η^2^ = 0.08, *f* = 0.30). The TC/HDL-C ratio increased after stress, with highest ratios immediately after stress cessation (main effect of stress: *F*(2.46, 135.38) = 6.62, *p* < 0.001, partial η^2^ = 0.11, *f* = 0.35) (see Figure 1C). Compared to baseline, post hoc testing revealed significant increases in TC/HDL-C ratio immediately (*p* = 0.017) and 10 min after stress (*p* = 0.028), whereas TG levels decreased immediately after stress cessation (*p* = 0.044).

### 3.3. Reactivity to the MIST in Hypertensives as Compared to Normotensives

#### 3.3.1. Baseline

At MIST baseline (see Table 2), hypertensives had higher TC/HDL-C ratio (*p* = 0.023) but did not differ in cortisol (*p* = 0.16) or other blood lipid (*p*-values ≥ 0.11) levels.

#### 3.3.2. Reactivity

**Figure 2:**
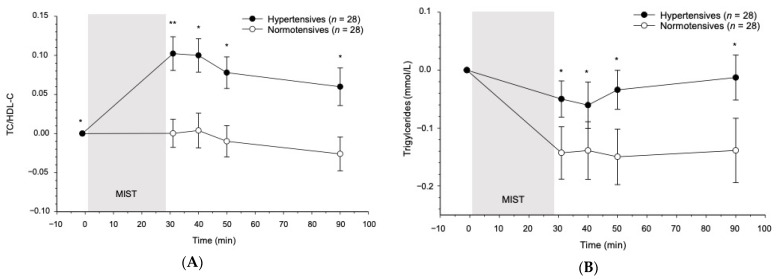
Blood lipid reactivity to the MIST in hypertensive and normotensive men. Values are means ± *SEM*. (**A**) MIST-induced TC/HDL-C ratio changes from baseline. (**B**) MIST-induced TG changes from baseline. * *p* < 0.05; ** *p* < 0.01.

In terms of blood lipids, hypertensives and normotensives significantly differed in the stress response of the TC/HDL-C ratio (interaction group-by-time: *F*(2.79, 145.41) = 4.09, *p* = 0.010, partial η^2^ = 0.07, *f* = 0.28). Hypertensives showed a higher TC/HDL-C ratio in response to stress compared with normotensive men (see Figure 2A and Table A2) at all post-stress measurements (+1 min: *p* = 0.009; +10 min: *p* = 0.010; +20 min: *p* = 0.010; +60 min: *p* = 0.011). Complementary analyses using MAP instead of group confirmed higher TC/HDL-C ratio stress reactivity with increasing MAP (interaction MAP-by-time: *F*(2.73, 141.76) = 2.25, *p* = 0.091, partial η^2^ = 0.04, *f* = 0.21). The two groups differed also in their stress-induced *TG* reactivity (interaction group-by-time: *F*(2.15, 111.59) = 3.02, *p* = 0.049, partial η^2^ = 0.06, *f* = 0.24) (see Figure 2B). Compared with normotensives, hypertensives showed higher TG levels (Figure 2B) (+1 min: *p* = 0.034; +10 min: *p* = 0.027; +20 min: *p* = 0.017; +60 min: *p* = 0.013) and thus a lower decline in TG levels after stress cessation. The reactivity difference in TG between hypertensives and normotensives was confirmed in complementary analyses using MAP as continuous dependent variable (interaction MAP-by-time: *F*(2.14, 111.37) = 4.36, *p* = 0.013, partial η^2^ = 0.08, *f* = 0.29). Stress-induced reactivity in TC (*p* = 0.71), in HDL-C (*p* = 0.65), and in LDL-C (*p* = 0.60) did not differ between groups. There were no group differences in terms of cortisol reactivity to the MIST (*p* = 0.12).

### 3.4. Prediction of CVD Risk Factors

MANOVA revealed that higher aggregated TC/HDL-C stress reactivity (AUC_G_) significantly predicted increased CVD risk in terms of increases in the measured CVD risk factors from baseline to follow-up (main effect in multivariate testing: *F*(4, 30) = 2.95, *p* = 0.036, partial η^2^ = 0.28, *f* = 0.62, Wilk’s Λ = 0.72). Complementary MANCOVA analyses controlling for age, MAP, and time between baseline and follow-up assessments as covariates confirmed the main effect of TC/HDL-C stress reactivity as a predictor of increased CVD risk over time (*F*(4, 27) = 3.35, *p* = 0.024, partial η^2^ = 0.33, *f* = 0.70, Wilk’s Λ = 0.67). Additional controlling for BMI, changes in BMI and MAP over time, or medication-intake at follow-up did not alter the significance of this main effect (*p*-values ≤ 0.029).

**Figure 3:**
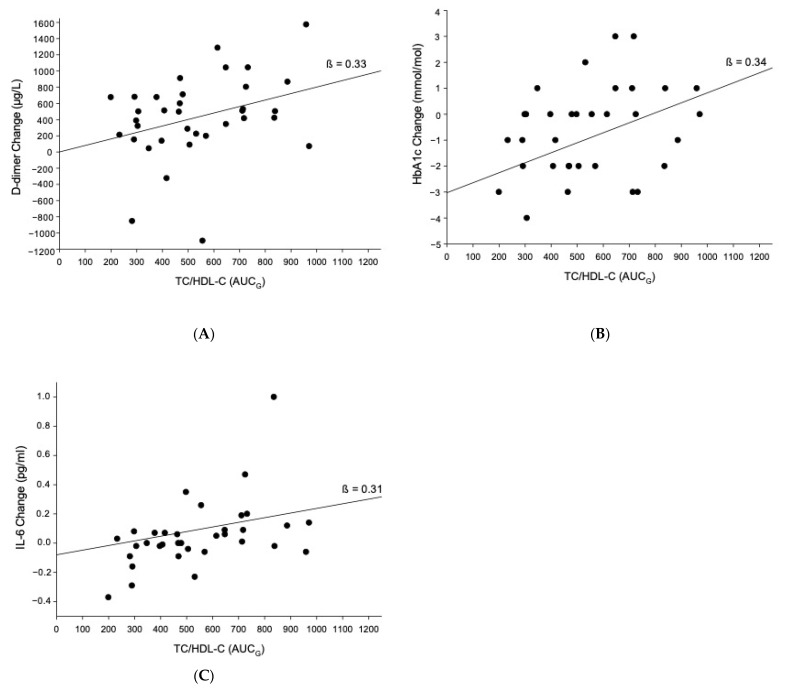
Higher aggregated TC/HDL-C stress reactivity (AUC_G_) significantly predicted higher increases from baseline to follow-up in CVD risk factors: (**A**) D-dimer (ß = 0.33, *p* = 0.050, Δ*R*^2^ = 0.11); (**B**) HbA1c (ß = 0.34, *p* = 0.046, Δ*R*^2^ = 0.12); (**C**) IL-6 (ß = 0.31, *p* = 0.075, Δ*R*^2^ = 0.09).

Post hoc testing revealed that higher blood lipid stress reactivity in terms of TC/HDL-C AUC_G_ predicted greater increases from baseline to follow-up in D-dimer (without control: ß = 0.33, *p* = 0.050, Δ*R*^2^ = 0.11, Figure 3A; controlling for the set of covariates: *p* = 0.11; additional controlling for medication intake, BMI, BMI change, or MAP change: *p*-values ≥ 0.09), HbA1c (without control: ß = 0.34, *p* = 0.046, Δ*R*^2^ = 0.12, Figure 3B; controlling for the set of covariates: ß = 0.32, *p* = 0.32, Δ*R*^2^ = 0.15; additional controlling for medication intake, BMI, BMI change, or MAP change: *p*-values ≥ 0.32), and IL-6 (without control: ß = 0.31, *p* = 0.075, Δ*R*^2^ = 0.09, Figure 3C; controlling for the set of covariates: ß = 0.45, *p* = 0.019, Δ*R*^2^ = 0.24 additional controlling for medication intake, BMI, BMI change, or MAP change: ß-values ≥ 0.43, *p*-values ≤ 0.026, Δ*R*^2^ ≥ 0.24). Prediction of higher TC/HDL-C increases was not significant (*p* = 0.57) but became of borderline significance when controlling for the set of covariates (ß = 0.23, *p* = 0.064, Δ*R*^2^ = 0.58; additional controlling for medication intake, BMI, BMI change, or MAP change: ß-values ≥ 0.25, *p*-values ≤ 0.073, Δ*R*^2^ ≥ 0.59).

## 4. Discussion

The main objective of our study was to investigate whether acute psychosocial stress induced by the MIST would relate to changes in blood lipid levels (i.e., TC, HDL-C, LDL-C, TG, and TC/HDL-C ratio) with expected reactivity differences between hypertensive and normotensive participants. We were further interested in the clinical relevance of blood lipid stress responses and tested whether TC/HDL-C stress reactivity would prospectively predict changes in CVD risk factors.

Our results confirm that stress induction by means of the MIST was successful, as indicated by salivary cortisol increases in line with previous MIST studies [22]. Moreover, we could extend the stress-provoking potential of the MIST for the first time to blood lipids. Independent of hemoconcentration, we observed significant increases in HDL-C, LDL-C, as well as in TC/HDL-C ratio scores in response to the MIST. The observed increases in atherogenic lipids are in line with previous studies using other potent stressors while correcting for hemoconcentration effects [15,16,17]. Interestingly, in our study, TG levels decreased after stress induction. Because triglycerides serve as energy reserves, we assume that the decrease in triglycerides after stress may represent a process of energy supply in reaction to stress-induced increased energy expenditure [38].

With respect to group differences, hypertensives showed a more pronounced rise in TC/HDL-C ratio scores and a slower decrease in TG levels in response to the MIST compared with normotensive participants. Complementary analyses using MAP confirmed the linear nature of this effect. The observed stress-related changes in atherogenic lipids resemble our previous observations in a TSST study [17]. We interpret our findings to mean that with increasing BP, hypertensives seem to be more vulnerable to acute psychosocial stress in terms of blood lipid responses. Group differences in stress-induced blood lipid reactivity might be elicited by the metabolic effect of catecholamine spillover in sympathetic nerve endings and from the adrenal medulla in response to acute psychosocial stress [17,39]. Increases in circulating catecholamines can induce lipolysis and release free fatty acids into the circulation [39]. With respect to cortisol analyzed in our study, stress induction by means of the MIST did not result in group differences between hypertensives and normotensives as observed in our previous TSST study [40]. We assume that the differences in stress-evoking components between the MIST and the TSST may account for this discrepancy. We performed the MIST in an MRT scanner, and the MRT experience may constitute part of the MIST stress experience that is not psychosocial in nature. Moreover, the direct contact with the experimenter is lower in the MRT scanner-based MIST as compared to the TSST with its permanent social confrontation. If the cortisol stress hyperreactivity observed in hypertensive participants mainly results from psychosocial stress aspects, their reactivity to the MIST might be comparably lower. At the same time, it is possible that normotensive participants may react stronger to the MIST, especially if performed within the potentially intimidating surrounding of an MRT scanner, as to the primarily psychosocial TSST.

Our prospective analyses were aimed to shed light on the supposed clinical relevance of blood lipid stress reactivity. Indeed, we found evidence for blood lipid stress reactivity in predicting future CVD risk in terms of increases over time in the intermediate biological risk factors HbA1c, D-dimer, and Il-6, and in part also in the TC/HDL-C ratio. Notably, one hypertensive participant started intake of cholesterol-lowering medication between baseline and follow-up assessment, and exclusion of this participant from analyses did not significantly change the observed prediction (data not shown). Given the observed higher blood lipid stress reactivity with increasing hypertension, our prospective findings suggest that blood lipid stress reactivity may play a role in mediating future CVD risk in hypertensives. We offer several possible explanations for these findings: (1) Atherosclerosis with its underlying process of chronic inflammation is central to CVD [41,42]. We speculate that increases in blood lipids, even if transient, may promote atherosclerosis by acutely forcing blood lipids into the arterial wall, thus adding to lipid accumulation in the intima-media of the carotid arteries. Increased lipid accumulation in the arterial wall of carotid arteries may relate to increased amounts of oxidized lipids, which in turn promote both inflammatory and atherothrombotic processes in atherosclerosis [41,42]. In line with such reasoning, we observed higher lipid stress reactivity to predict higher prospective IL-6 and fibrin D-dimer increases. At the same time, both IL-6 and fibrin D-dimer are independent CVD risk factors that can further promote atherosclerotic processes [25,26,43]. (2) Our borderline significant finding of blood lipid stress reactivity independently predicting increased TC/HDL-C ratio over time confirms a previous study by Steptoe et al. [24]. Extending these prior results, our data suggest that this association seems to be independent of hemoconcentration effects. In line with the allostatic load model, we speculate that repeated stressful experiences may accumulate over time to result in chronic elevations of blood lipid levels [44], but the precise underlying mechanism warrants further investigation. (3) Diabetic dyslipidemia is a well-known phenomenon, especially in the context of the metabolic syndrome clustering increased BP, high blood sugar, and dyslipidemia [45]. However, potential mechanisms underlying our finding of blood lipid stress responses to predict increases in HbA1c as an indicator of increased blood sugar over time are unclear. Notably, our observed associations were not independent of confounders, in particular of basal MAP, suggesting that basal BP effects may play a role in modulating the observed HbA1c increases.

Limitations of our study are the relatively small number of participants due to the experimental nature of our study and the comparably high drop-out rate that we mainly attribute to organizational reasons. While the participant number for our stress study was based on our a priori sample size calculations allowing to detect effects of small-to-medium effect sizes, the reduced sample size available for the prospective analyses only allowed to detect medium-to-large effects. We therefore cannot rule out the possibility that the study was underpowered to detect smaller effects and that our results might be biased. Notably, given our follow-up sample size, we only allowed for a maximum of four confounders in follow-up analyses [46]. Although participants who dropped out did not substantially differ in their characteristics at baseline from those completing the follow-up assessment, there were more drop-outs in the hypertensive group. Furthermore, eight participants started medication intake between baseline and follow-up assessment, which could possibly influence prospective outcomes, although we controlled for potential medication effects. Finally, the generalizability of our results is limited to middle-aged normotensive and essentially hypertensive men.

## 5. Conclusions

Taken together, we observed significant changes in blood lipid levels in reaction to stress induction by means of the MIST. Blood lipid levels were higher after stress in hypertensive individuals compared with normotensive controls and also with increasing MAP. As the aggregated TC/HDL-C stress reaction predicted CVD risk factors several years later, blood lipid stress reactivity might be a potential mechanism contributing to a poorer cardiovascular health in hypertensives. Future studies are needed to replicate our cross-sectional and in particular prospective findings in larger sample sizes including women and clinical populations. Subsequent studies could further investigate whether specific medication (e.g., statin therapy) could attenuate the effects of stress on lipid levels. Moreover, the mechanisms underlying the observed stress-reactivity differences between hypertensives and normotensives and the prospective associations between blood lipid stress reactivity and CVD risk warrant further investigation.

## Figures and Tables

**Table 1:** Comparison of baseline group characteristics between hypertensive and normotensive participants.

	Hypertensives (*n* = 28)	Normotensives (*n* = 28)	*p*
Age (years)	49.82 ± 2.04 (25–74)	49.75 ± 2.22 (28–74)	0.98
BMI (kg/m^2^)	28.29 ± 0.64 (22.67–35.25)	24.99 ± 0.55 (19.78–30.85)	<0.001 ***
Home BP (mmHg)			
Home MAP	106.85 ± 1.17 (95.61–121.50), *n* = 26	87.21 ± 0.92 (75.06–94.72)	<0.001 ***
Home SBP	144.83 ± 1.82 (125.67–162.33), *n* = 26	121.08 ± 1.19 (105.17–130.50)	<0.001 ***
Home DBP	87.85 ± 1.28 (78.33–103.00), *n* = 26	70.27 ± 1.011 (60.00–79.33)	<0.001 ***
Study BP (mmHg)			
Study MAP	115.96 ± 1.84 (103.50–139.89)	93.88 ± 1.41 (75.33–104.56)	<0.001 ***
Study SBP	153.70 ± 2.65 (129.33–189.67)	127.39 ± 1.67 (109.33–139.67)	<0.001 ***
Study DBP	97.08 ± 1.58 (84.67–115.00)	77.13 ± 1.46 (58.33–87.67)	<0.001 ***
TG (mmol/L)	1.52 ± 0.12 (0.77–3.76), *n* = 27	1.10 ± 0.09 (0.36–2.35), *n* = 27	0.007 **
TC (mmol/L)	5.67 ± 0.16 (4.11–7.18), *n* = 27	5.22 ± 0.23 (3.30–7.79), *n* = 27	0.12
LDL-C(mmol/L)	3.91 ± 0.16 (2.33–5.19), *n* = 27	3.48 ± 0.22 (1.73–6.53), *n* = 27	0.11
HDL-C (mmol/L)	1.43 ± 0.07 (0.93–2.22), *n* = 27	1.59 ± 0.06 (0.92–2.21), *n* = 27	0.11
LDL-C/HDL-C	2.88 ± 0.16 (1.32–4.32), *n* = 27	2.26 ± 0.16 (1.05–4.35), *n* = 27	0.010 *
TC/HDL-C	4.14 ± 0.19. (2.38–5.77), *n* = 27	3.41 ± 0.19 (2.01–5.49), *n* = 27	0.009 **
HbA1c (mmol/mol)	35.59 ± 0.82 (26–43), *n* = 27	36.59 ± 0.57 (31–42), *n* = 27	0.28
D-dimer (µg/L)	375.37 ± 40.73 (45–955), *n* = 27	440.82 ± 72.56 (45–1616)	0.77
IL-6 (pg/mL)	0.63 ± 0.07 (0.16–1.85), *n* = 27	0.37 ± 0.03 (0.15–0.76)	<0.001 ***
Time between baseline and follow-up assessments (months)	40.75 ± 2.14 (28–61)	39.33 ± 1.81 (28–63)	0.62
Creatinine (μmol/L)	80.48 ± 1.63 (66–93), *n* = 27		
Sodium (mmol/L)	140.39 ± 0.50 (135–145), *n* = 23		
Calcium (mmol/L)	2.40 ± 0.02 (2.17–2.58), *n* = 23		
Potassium (mmol/L)	4.11 ± 0.04 (3.9–4.7), *n* = 23		

Notes. Values are means ± *SEM* (range); BMI = body mass index; BP = blood pressure; MAP = mean arterial blood pressure; DBP = diastolic blood pressure; SBP = systolic blood pressure; TG = triglycerides; TC = total cholesterol; LDL-C = low-density lipoprotein cholesterol; HDL-C = high-density lipoprotein cholesterol; HbA1c = hemoglobin A1c; IL-6 = interleukin-6. Deviating participant numbers are indicated. * *p* < 0.05; ** *p* < 0.01; *** *p* < 0.001.

**Table 2:** Comparison of MIST baseline levels of cortisol and blood lipids in hypertensives and normotensives.

	Hypertensives (*n* = 28)	Normotensives (*n* = 28)	*p*
Cortisol (nmol/L)	7.22 ± 0.92 (1.83–17.85)	5.21 ± 0.50 (1.61–12.50)	0.16
HDL-C (mmol/L)	1.25 ± 0.06 (0.87–1.93)	1.35 ± 0.05 (0.86–1.83)	0.20
LDL-C (mmol/L)	3.31 ± 0.15 (1.63–4.51)	2.95 ± 0.16 (1.36–5.37)	0.11
TG (mmol/L)	1.66 ± 0.14 (0.61–3.61)	1.38 ± 0.11 (0.35–2.87)	0.12
TC/HDL-C	4.14 ± 0.19 (2.46–6.29)	3.54 ± 0.17 (2.13–5.65)	0.023 *

Notes. Values are means ± *SEM* (range); HDL-C = high-density lipoprotein cholesterol; LDL-C = low-density lipoprotein cholesterol; TG = triglycerides; TC = total cholesterol. * *p* < 0.05.

## Data Availability

Not available.

## Appendix A

**Table A1:** Comparison of baseline group characteristics between participants completing follow-up assessment and drop-out participants.

	Follow-Up (*n* = 35)	Drop-Out (*n* = 21)	*p*
Age (years)	50.20 ± 1.91 (28–74)	49.10 ± 2.45 (25–69)	0.72
BMI (kg/m^2^)	26.37 ± 0.62 (19.78–34.58)	27.08 ± 0.73 (22.67–35.25)	0.47
Home BP (mmHg)			
Home MAP	96.17 ± 2.14 (75.06–121.50)	97.34 ± 2.21 (82.39–110.94)	0.66
Home SBP	131.04 ± 2.63 (105.17–162.33)	135.03 ± 2.75 (115.17–154.50)	0.29
Home DBP	78.77 ± 1.90 (60–103)	78.68 ± 2.25 (62.83–91.17)	0.98
Study BP (mmHg)			
Study MAP	103.73 ± 2.58 (75.33–139.89)	106.90 ± 2.57 (79.83–126.56)	0.42
Study SBP	139.39 ± 3.18 (109.33–189.67)	142.48 ± 3.44 (112.5–166.33)	0.53
Study DBP	85.90 ± 2.37 (58.33–115)	89.11 ± 2.32 (63.50–108.00)	0.37
TG (mmol/L)	1.38 ± 0.11 (0.36–3.76)	1.15 ± 0.07 (0.71–1.88)	0.15
TC (mmol/L)	5.36 ± 0.16 (3.30–7.69)	5.60 ± 0.27 (3.90–7.79)	0.41
LDL-C (mmol/L)	3.61 ± 0.14 (1.73–5.69)	3.82 ± 0.27 (2.09–6.53)	0.45
HDL-C (mmol/L)	1.49 ± 0.06 (0.92–2.21)	1.58 ± 0.09 (1.08–2.22)	0.35
LDL-C/HDL-C	2.57 ± 0.14 (1.05–3.97)	2.57 ± 0.23 (1.07–4.35)	0.98
TC/HDL-C	3.78 ± 0.17 (2.01–5.49)	3.72 ± 0.25 (2.03–5.77)	0.79
HbA1c (mmol/mol)	36.26 ± 0.63 (28–43)	35.79 ± 0.83 (26–40)	0.67
D-dimer (µg/L)	416.43 ± 59.05 (45–1616)	395.15 ± 52.52 (45–955)	0.98
IL-6 (pg/mL)	0.47 ± 0.04 (0.15–1.25)	0.55 ± 0.08 (0.21–1.85)	0.37

Notes. Values are means ± *SEM* (range); BMI = body mass index; BP = blood pressure; MAP = mean arterial blood pressure; DBP = diastolic blood pressure; SBP = systolic blood pressure; TG = triglycerides; TC = total cholesterol; LDL-C = low-density lipoprotein cholesterol; HDL-C = high-density lipoprotein cholesterol; HbA1c = hemoglobin A1c; IL-6 = interleukin-6.

**Table A2:** Blood lipid reactivity in hypertensive and normotensive participants.

	Hypertensives (*n* = 28)	Normotensives (*n* = 28)	*p*
TC/HDL-C			
−1 min	4.14 ± 0.19 (2.46–6.29)	3.54 ± 0.17 (2.13–5.65)	0.023 *
+1 min	4.24 ± 0.20 (2.45–6.28)	3.54 ± 0.17 (2.15–5.51)	0.009 **
+10 min	4.24 ± 0.20 (2.44–6.52)	3.54 ± 0.17(2.16–5.62)	0.010 *
+20 min	4.22 ± 0.20 (2.43–6.26)	3.53 ± 0.17 (2.17–5.53)	0.010 *
+60 min	4.20 ± 0.20 (2.42–6.14)	3.51 ± 0.17 (2.12–5.58)	0.011 *
TG (mmol/L)			
−1 min	1.66 ±0.14 (0.61–3.61)	1.38 ± 0.11 (0.35–2.87)	0.121
+1 min	1.61 ± 0.13 (0.62–3.44)	1.24 ± 0.09 (0.37–2.21)	0.022 *
+10 min	1.60 ± 0.13 (0.67–3.44)	1.24 ± 0.10 (0.39–2.30)	0.027 *
+20 min	1.63 ± 0.13 (0.68–3.22)	1.23 ± 0.09 (0.39–2.30)	0.014 *
+60 min	1.65 ± 0.13 (0.71–3.41)	1.24 ± 0.09 (0.39–2.29)	0.013 *
TC (mmol/L)			
−1 min	4.97 ± 0.17 (3.16–6.53)	4.63 ± 0.18 (2.69–7.01)	0.18
+1 min	4.95 ± 0.18 (3.29–6.60)	4.66 ± 0.18 (2.80–7.05)	0.25
+10 min	4.99 ± 0.19 (3.20–7.13)	4.76 ± 0.19 (2.87–7.21)	0.39
+20 min	5.03 ± 0.19 (3.26–7.16)	4.66 ± 0.18 (2.77–7.15)	0.16
+60 min	5.03 ± 0.19 (3.20–6.93)	4.73 ± 0.19 (2.79–7.41)	0.26
HDL (mmol/L)			
−1 min	1.25 ± 0.06 (0.87–1.93)	1.35 ± 0.05 (0.86–1.83)	0.20
+1 min	1.21 ± 0.05 (0.80–1.96)	1.35 ± 0.05 (0.83–1.82)	0.06
+10 min	1.23 ± 0.07 (0.67–2.02)	1.38 ± 0.05 (0.86–1.78)	0.09
+20 min	1.24 ± 0.06 (0.87–1.97)	1.36 ± 0.05(0.83–1.79)	0.14
+60 min	1.24 ± 0.06 (0.86–2.01)	1.38 ± 0.05 (0.86–1.88)	0.08
LDL (mmol/L)			
−1 min	3.31 ± 0.15 (1.63–4.51)	2.95 ± 0.16 (1.36–5.37)	0.11
+1 min	3.30 ± 0.16 (1.71–4.61)	2.92 ± 0.18 (0.57–5.48)	0.13
+10 min	3.31 ± 0.16 (1.67–5.00)	3.06 ± 0.17 (1.43–5.59)	0.27
+20 min	3.36 ± 0.16 (1.73–4.99)	3.00 ± 0.16 (1.39–5.58)	0.13
+60 min	3.38 ± 0.16 (1.72–5.04)	3.06 ± 0.17 (1.42–5.64)	0.18

Notes. Values are means ± *SEM* (range); TC = total cholesterol; HDL-C = high-density lipoprotein cholesterol; TG = triglycerides; LDL-C = low-density lipoprotein cholesterol. * *p* < 0.05; ** *p* < 0.01.
